# Agitation Following Severe Traumatic Brain Injury Is a Clinical Sign of Recovery of Consciousness

**DOI:** 10.3389/fsurg.2021.627008

**Published:** 2021-04-21

**Authors:** Zhe Wang, Nathan J. Winans, Zirun Zhao, Megan E. Cosgrove, Theresa Gammel, Jordan R. Saadon, Racheed Mani, Bharadwaj Ravi, Susan M. Fiore, Charles B. Mikell, Sima Mofakham

**Affiliations:** ^1^Department of Neurological Surgery, Renaissance School of Medicine at Stony Brook University, Stony Brook, NY, United States; ^2^Department of Neurological Surgery, Columbia University Medical Center, New York, NY, United States; ^3^Department of Neurobiology and Behavior, Renaissance School of Medicine at Stony Brook University, Stony Brook, NY, United States

**Keywords:** severe traumatic brain injury, coma, posttraumatic agitation, recovery of consciousness, antipsychotics

## Abstract

**Objective:** Severe traumatic brain injury (sTBI) often results in disorders of consciousness. Patients emerging from coma frequently exhibit aberrant behaviors such as agitation. These non-purposeful combative behaviors can interfere with medical care. Interestingly, agitation is associated with arousal and is often among the first signs of neurological recovery. A better understanding of these behaviors may shed light on the mechanisms driving the return of consciousness in sTBI patients. This study aims to investigate the association between posttraumatic agitation and the recovery of consciousness.

**Methods:** A retrospective chart review was conducted in 530 adult patients (29.1% female) admitted to Stony Brook University Hospital between January 2011 and December 2019 with a diagnosis of sTBI and Glasgow Coma Scale (GCS) ≤8. Agitation was defined as a Richmond Agitation Sedation Scale (RASS) > +1, or any documentation of equivalently combative and violent behaviors in daily clinical notes. The ability to follow verbal commands was used to define the recovery of consciousness and was assessed daily.

**Results:** Of 530 total sTBI patients, 308 (58.1%) survived. Agitation was present in 169 of all patients and 162 (52.6%) of surviving patients. A total of 273 patients followed commands, and 159 of them developed agitation. Forty patients developed agitation on hospital arrival whereas 119 developed agitation later during their hospital course. Presence of in-hospital agitation positively correlated with command-following (*r* = 0.315, *p* < 0.001). The time to develop agitation and time to follow commands showed positive correlation (*r* = 0.485, *p* < 0.001). These two events occurred within 3 days in 54 (44.6%) patients, within 7 days in 81 (67.8%) patients, and within 14 days in 96 (80.2%) patients. In 71 (59.7%) patients, agitation developed before command-following; in 36 (30.2%) patients, agitation developed after command-following; in 12 (10.1%) patients, agitation developed on the same day as command-following.

**Conclusion:** Posttraumatic agitation in comatose patients following sTBI is temporally associated with the recovery of consciousness. This behavior indicates the potential for recovery of higher neurological functioning. Further studies are required to identify neural correlates of posttraumatic agitation and recovery of consciousness after sTBI.

## Introduction

Severe traumatic brain injury (sTBI) often results in disorders of consciousness (1). Patients in comatose states can exhibit aberrant motor behaviors, characterized by hyperactivity, non-purposeful movement, and combativeness that are disruptive to patient care (2, 3). Agitation is frequently encountered in sTBI patients in the hospital setting (4, 5). The prevalence of in-hospital agitation in patients following sTBI is between 10 and 90% (2, 5). This large variability can be explained by differences in diagnostic criteria and under- or over-recognition of these behaviors (6, 7). Interestingly, these behaviors represent fluctuations of consciousness during periods when patients reemerge from posttraumatic coma (8). Thus, a thorough understanding of the mechanisms driving agitation is imperative for patient safety and facilitation of recovery-directed treatment for sTBI.

The underlying mechanisms of posttraumatic agitation are poorly understood. Several theories have proposed that this behavior is likely secondary to alterations in brain metabolism, dysregulation of neural transmission, and remodeling of neural network (9). Dysregulation of dopaminergic neurotransmission, for example, often correlates with behavioral changes after neurologic insults (10). Pharmacotherapies, such as amantadine, have been used to facilitate arousal by stimulating the dopaminergic pathway (11). Randomized controlled clinical trials have shown that amantadine promotes cognitive recovery in sTBI patients who are in prolonged coma (12). Amantadine has also been reported to increase the incidence of in-hospital agitation when used in the early stage after TBI (13). These findings suggest that agitation and arousal likely share the same neurological pathways and are associated with the recovery of consciousness.

The first-line treatment for agitation is by providing patient-centered care through environmental changes, such as sufficient pain relief, light-dark cycle simulation, and nursing staff consistency (14). Antipsychotics are commonly used to manage agitation in critically ill patients who are unresponsive to conservative measures (15). Both typical and atypical antipsychotics suppress dopaminergic neurotransmission and have been used as prophylaxis and/or treatment for confusion and agitation, but recent studies suggest that the efficacy is limited (16). Importantly, the safety of using antipsychotics in sTBI patients is not known with certainty (17–19). Retrospective clinical studies have shown that using antipsychotics to treat posttraumatic delirium can impair cognitive recovery (18). Evidence from TBI animal models suggests that both typical and atypical antipsychotics hinder recovery from TBI-related deficits (20–22). These results suggest that dopaminergic suppression interferes with arousal and consequently recovery of consciousness. Currently, two randomized controlled clinical trials are being conducted to evaluate the safety and efficacy of antipsychotics in sTBI patients in rehabilitation settings (23, 24). Results from these studies can shed light on the underlying mechanism of agitation during the early stage of recovery from sTBI.

Given the clinical significance of posttraumatic agitation in patients emerging from disorders of consciousness, early recognition and appropriate management will benefit neurological outcomes. The balance between promoting arousal in early stage of recovery from sTBI and the risk of developing agitation is often patient-specific and is difficult to achieve. Understanding the clinical features of posttraumatic agitation and its relationship to arousal can establish patient-specific treatment plans, as these goal-directed behaviors are often among the first signs of returning of consciousness. In the present study, we evaluate the relationship between posttraumatic agitation and the recovery of consciousness in comatose patients following sTBI in the acute hospital setting. By identifying the association between the two, we emphasize the clinical importance of agitated behaviors, and advise prudent clinical management.

## Methods

### Subjects

Adult patients (age ≥18 at time of injury) admitted to Stony Brook University Hospital, a level 1 trauma center in Long Island, New York, from January 2011 to December 2019 with a clinical diagnosis of sTBI and Glasgow Coma Scale (GCS) ≤ 8 were included in the analysis. All study procedures were approved by the Stony Brook University Committee on Research in Human Subjects, in accordance with federal guidelines.

### Data Collection

Data was collected retrospectively from individual patient charts. Admission profiles such as age, gender, GCS, injury severity score (ISS), and mechanisms of injury were used to describe baseline patient characteristics. When available, non-contrast CT scans were reviewed and evaluated using the Rotterdam head CT scoring system (25). Levels of agitation and sedation were assessed hourly by nursing stuff using the Richmond Agitation Sedation Scale (RASS) (6). The RASS is a validated tool used to guide sedation therapy in hospitalized patients. The scale uses a rating severity from−5 (unarousable) to +4 (combative). RASS > +1, or documentation of agitated behaviors that were equivalently combative was used to define agitation in our cohort. These behaviors were characterized by non-purposeful movement, patient-ventilator dyssynchrony, or self-harming behaviors, and required immediate medical intervention, such as higher dose of sedation or physical restraints. The ability to follow verbal commands (GCS motor score = 6) was used to define recovery of consciousness and was evaluated daily by clinicians. The date of first reported agitation and the date of following commands were used to analyze correlation between the two. Length of hospital stay (LOS), time to follow commands (TFC), discharge GCS, and discharge location were used as outcome measures.

### Statistical Analysis

Descriptive statistics are reported as percentage, mean, or standard deviation wherever appropriate. Extreme values (≥3 standard deviations from the mean of the original dataset) were removed for more accurate data representation. For between-group comparisons, two-tailed Student's *t*-test and Chi-square were used to test statistical significance of continuous and categorical variables, respectively. Statistical significance was defined as *p* < 0.05. Statistical analysis was performed using SPSS, version 26 (IBM Corp).

## Results

### Patient Characteristics

**Figure 1:**
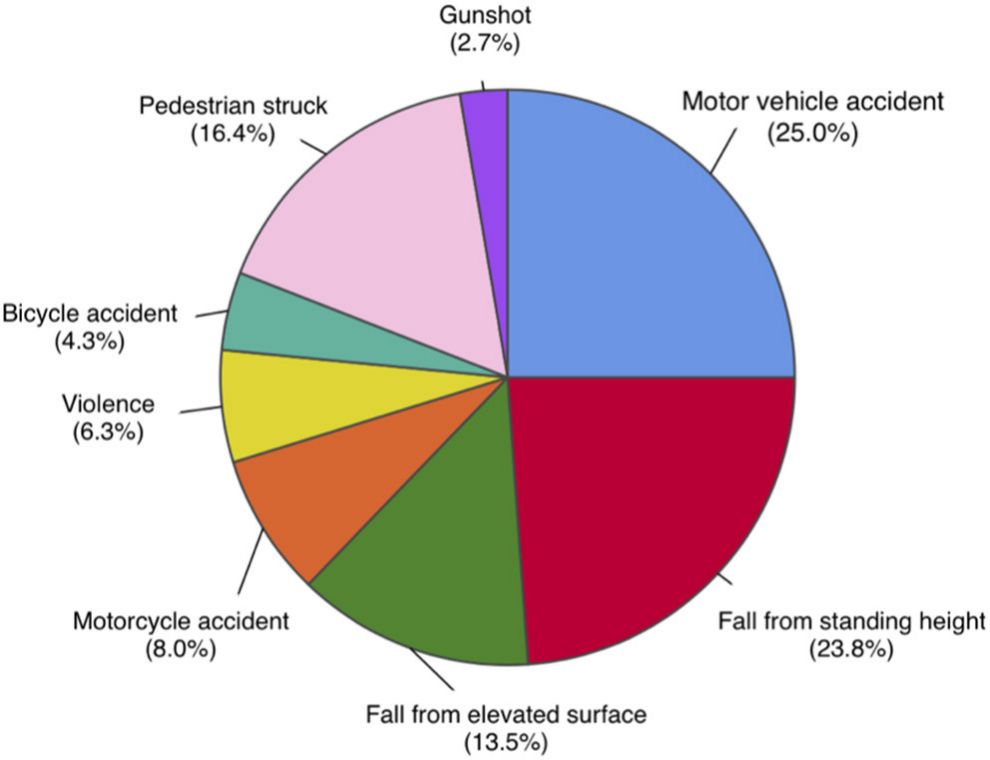
Distribution of mechanisms of injury in total study population, *n* = 530. Road traffic accidents, including motor vehicle, motorcycle, bicycle accidents, and pedestrian struck, are most common mechanisms of injury, accounting for a total of 282 (53.7%) cases. Fall from elevated and standing heights is second most common, accounting for 196 (37.3%) cases. Other mechanisms include violence and gunshot wounds.

A total of 530 patients (29.1% female) were included in this study based on the eligibility criteria (Table 1). The most common cause of injury was road traffic accidents including motor vehicle, motorcycle, bicycle accidents, and pedestrian struck, accounting for a total of 282 (53.7%) cases. Fall from standing and elevated heights account for 196 (37.3%) cases. Other mechanisms of injury included violence and gunshot wounds (Figure 1). Of the 530 patients, 222 (41.9%) patients died as a result of their injuries. Of the 308 surviving patients, the average age was 44.8 (SD 19.6) years, ranging from 18 to 94 years old. The mean GCS on admission was 4.6 (SD 1.9), the mean injury severity score (ISS) was 27.9 (SD 13.0), and the mean Rotterdam score was 2.9 (SD 1.0). The mean length of hospital stay (LOS) was 34.4 (SD 36.3) days, ranging from 2 to 245 days. Among all surviving patients, 273 (88.6%) individuals recovered consciousness by following verbal commands during their hospitalization. The mean time to follow commands (TFC) was 10.1 (SD 12.3) days, ranging from 1 to 72 days. A majority of patients were discharged to rehabilitation facilities (63.0%) or home (26.6%), and the rest to nursing home/hospice (8.1%) or another hospital (2.3%). Most patients had a favorable course of recovery and were discharged with a significantly higher GCS compared to that on admission. Discharge GCS 13–15 was seen in 249 (80.8%) patients, GCS 9–12 seen in 33 (10.7%) patients, and GCS 3–8 seen in 26 (8.5%) patients (Table 2).

**Table 1:** Patient characteristics of total study population, *n* = 530.

**Total number of patients**	***n* = 530**
Mean age (years)	49.6 (SD 21.9)
Sex	
Male	376 (70.9%)
Female	154 (29.1%)
Mean GCS on admission	4.3 (SD 1.8)
Mean ISS	30.6 (SD 13.7)
Mean Rotterdam (n = 483)	3.4 (SD 1.3)
In-hospital mortality	222 (42.0%)
No. patients with in-hospital agitation	169 (31.9%)
No. patients with command-following	277 (52.3%)

*GCS, Glasgow Coma Scale; ISS, Injury severity score; LOS, Length of hospital stay; TFC, Time to follow commands*.

**Table 2:** Patient characteristics and clinical outcomes of surviving agitated and non-agitated patients, *n* = 308.

**Demographics**	**All surviving (*n* = 308)**	**Agitated (*n* = 162)**	**Not agitated (*n* = 146)**	***p*-value**
Mean age (years)	44.8 (SD 19.6)	40.7 (SD 17.7)	49.3 (SD 20.6)	**<0.001**
Sex				
Male	230 (74.7%)	129 (56.1%)	101 (43.9%)	**0.035**
Female	78 (25.3%)	33 (42.3%)	45 (57.7%)	
Mean GCS on admission	4.6 (SD 1.9)	4.7 (SD 1.9)	4.5 (SD (1.8)	0.25
Mean ISS	27.9 (SD 13.0)	27.9 (SD 13.2)	27.9 (SD 12.9)	0.97
Mean Rotterdam (*n* = 304)	2.9 (SD 1.0)	2.9 (SD 0.9)	3.0 (SD 1.1)	0.30
Mechanism of Injury				
Motor vehicle accident	86 (28.0%)	52 (31.7%)	36 (24.6%)	0.38
Fall from standing	67 (21.8%)	31 (19.3%)	36 (24.6%)	
Fall from elevated height	36 (11.7%)	19 (11.8%)	17 (11.6%)	
Motorcycle	27 (8.8%)	15 (9.3%)	12 (8.2%)	
Violence	25 (8.1%)	14 (8.7%)	11 (7.5%)	
Bicycle	16 (5.2%)	7 (4.3%)	9 (6.2%)	
Pedestrian struck	45 (14.7%)	21 (13.0%)	24 16.4%)	
Gunshot	2 (0.7%)	1 (0.6%)	1 (0.7%)	
Unwitnessed	3 (1.0%)	2 (1.2%)	1 (0.7%)	
Pupil Reactivity				
Bilateral reactive	237 (76.9%)	127 (78.4%)	110 (75.3%)	0.44
Bilateral non-reactive and constricted	30 (9.7%)	15 (9.3%)	15 (10.3%)	
Bilateral non-reactive and dilated	13 (4.2%)	8 (4.9%)	5 (3.4%)	
Single fixed and dilated or constricted	28 (9.1%)	12 (7.4%)	16 (11.0%)	
Mean LOS (days)	34.4 (SD 36.3)	34.1 (SD 32.1)	34.8 (SD 40.7)	0.86
95% confidence interval for mean		29.1–39.1	28.1–41.6	
Median		25.0	23.0	
Patients with command-following (*n* = 273)		*n* = 159	*n* = 114	
Mean TFC (days)	10.1 (SD 12.3)	10.6 (SD 11.8)	9.3 (SD 12.8)	0.41
95% confidence interval for mean		8.7–12.5	7.0–11.7	
Median		7.0	5.0	
Discharge GCS				
13–15	249 (80.8%)	147 (90.7%)	102 (70.0%)	**<0.001**
9–12	33 (10.7%)	9 (5.6%)	24 (16.4%)	
3–8	26 (8.5%)	6 (3.7%)	20 (13.7%)	
Discharge location				
Home	82 (26.6%)	49 (30.2%)	33 (22.6%)	**0.010**
Rehabilitation	194 (63.0%)	103 (63.6%)	91 (62.3%)	
Nursing home or hospice	25 (8.1%)	6 (3.7%)	19 (13.0%)	
Other hospital	7 (2.3%)	4 (2.5%)	3 (2.1%)	

*GCS, Glasgow Coma Scale; ISS, Injury severity score; LOS, Length of hospital stay; TFC, Time to follow commands*.

*Bold texts highlight the statistical significance*.

### Characteristics of Agitated Patients

**Figure 2:**
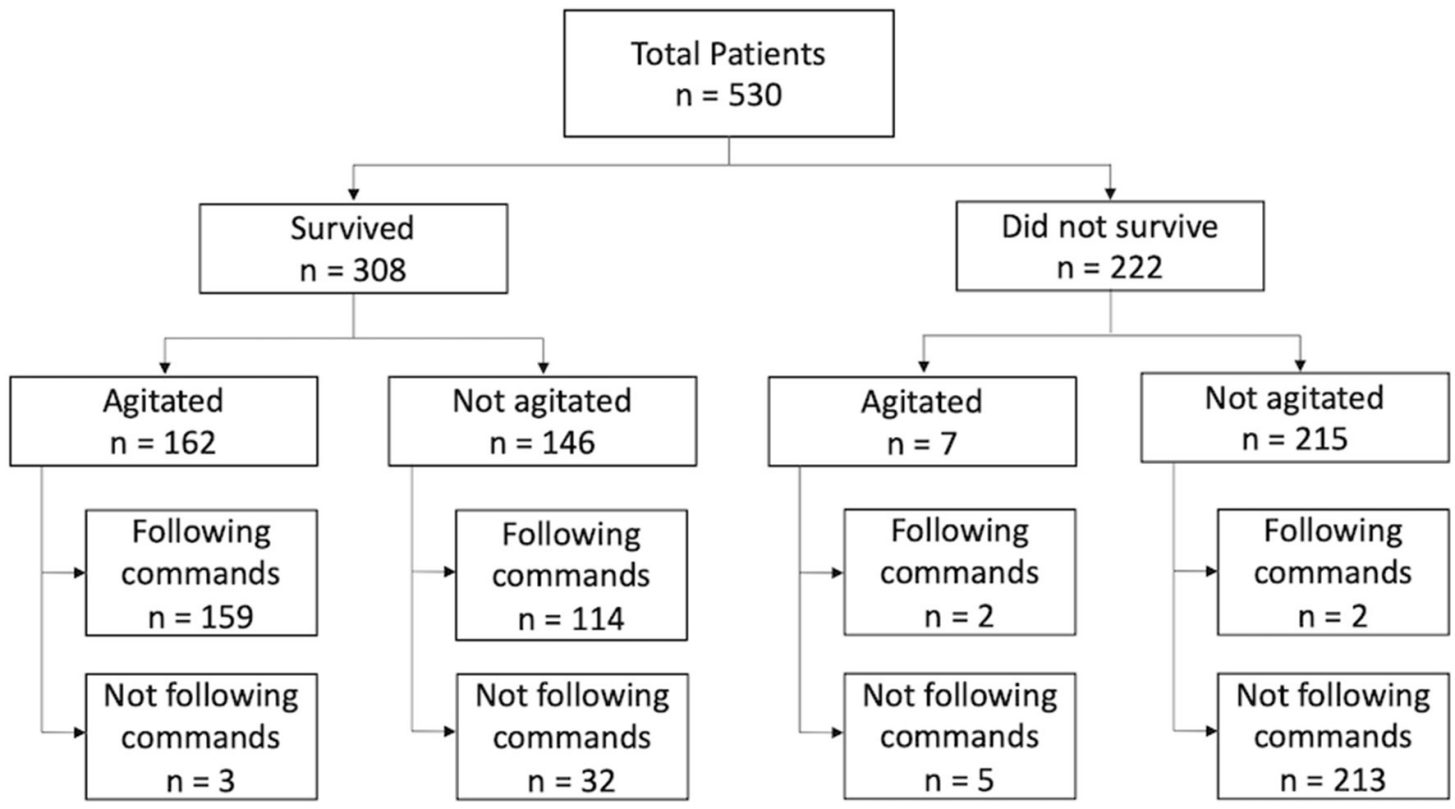
Flowchart of total study population, *n* = 530. Survival status was determined by retrospective chart review. Seven patients developed agitation but subsequently expired due to failure to wean off mechanical ventilation.

Agitation was observed in 162 (52.6%) of 308 surviving patients, and 159 of these individuals followed commands (Figure 2). The agitated cohort was on average 8.6 years younger than the non-agitated one (40.7 vs. 49.3 years, *p* < 0.001, Table 2). Males were more likely to develop agitation than females (56.1 vs. 42.3%, *p* = 0.035). There were no significant differences between agitated and non-agitated patients in admission GCS (4.7 vs. 4.5, *p* = 0.25), ISS (27.9 vs. 27.9, *p* = 0.97), and Rotterdam scores (2.9 vs. 3.0, *p* = 0.30), and pupil reactivity (*p* = 0.44). The mechanisms of injury have similar distributions between agitated and non-agitated patients, with road traffic accidents being the most common, followed by falls. A larger proportion of agitated patients were discharged with GCS 13–15 compared to the non-agitated ones (90.7 vs. 70.0%, *p* < 0.001, Table 2). Agitated patients were more frequently discharged home or to rehabilitation facilities, whereas a larger proportion of non-agitated patients were discharged to nursing home or hospice (*p* = 0.010). There were no significant differences in LOS (34.1 vs. 34.8 days, *p* = 0.86) or TFC (10.6 vs. 9.3 days, *p* = 0.41) between agitated and non-agitated patients (Table 2).

### Agitation Correlates With Recovery of Consciousness

**Figure 3:**
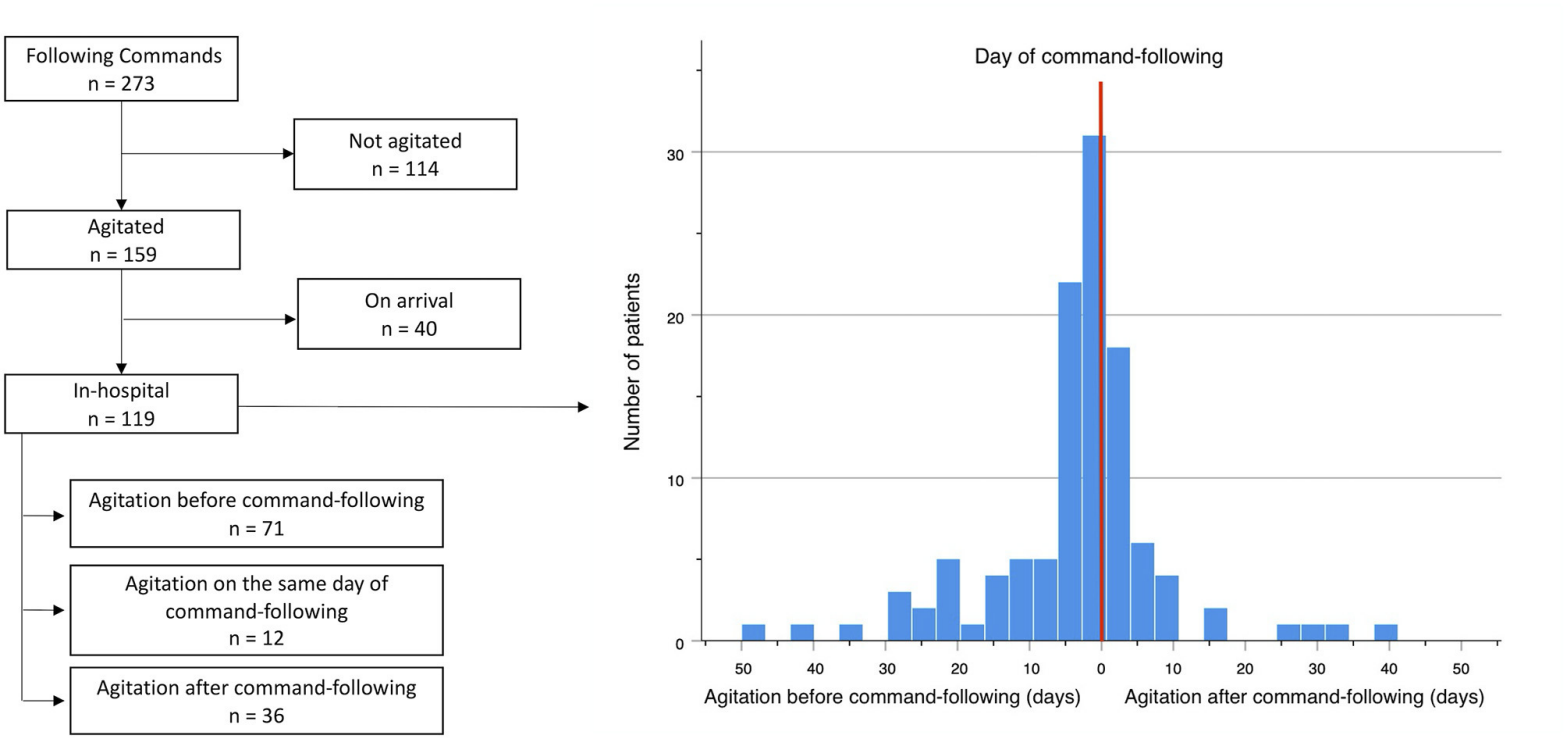
Temporal association between onset of agitation and command-following, *n* = 119. Red line represents the day the patient begins following commands. X-axis shows numbers of days from agitation onset to command-following. Left to the red line represents days from agitation to command-following. Right to the red line represents days from command following to agitation. Y-axis corresponds to the number of patients.

The ability to follow commands (GCS = 6) was achieved in 273 of all surviving patients, and agitation was observed in 159 (58.2%) of them (Figure 3). A positive correlation was observed between the presence of agitation and that of command-following (*r* = 0.315, *p* < 0.001). Time to develop agitation and time to follow commands was also positively correlated (*r* = 0.485, *p* < 0.001). Of these patients, 40 (24.7%) showed signs of agitation upon arrival to the hospital, whereas 119 (75.3%) patients arrived in a comatose state and developed agitation later during their hospital course. In these 119 patients, a temporal association was observed between the time of agitation onset and time to follow commands (Figure 3). In 54 (44.6%) of these patients, agitation and command-following were observed within 3 days of each other; in 81 (67.8%) patients, these two events occurred within 7 days; and in 96 (80.7%) patients, co-occurrence was observed within 14 days. Among these 119 patients, 71 (59.7%) developed signs of agitation before command-following by an average of 9.4 (SD 10.3) days, ranging from 1 to 47 days (95% CI: 6.9–11.8 days); 36 (30.2%) patients developed agitation after command-following by an average of 7.8 (SD 9.8) days, ranging from 1 to 40 days (95% CI: 4.4–11.2 days); and 12 (10.1%) patients developed agitation on the same day as command-following (Table 3).

**Table 3:** Association between agitation onset and command-following, *n* = 119.

**Total number of in-hospital agitation**	***n* = 119**	
Agitation before command-following	*n* = 71	
Mean (SD), days	9.4 (10.3)	
95% confidence interval	6.9–11.8	
Median	5.0	
Agitation After command-following	*n* = 36	
Mean (SD), days	7.8 (9.8)	
95% confidence interval	4.4–11.2	
Median	4.0	
Agitation same day as command-following	*n* = 12	
**Co-occurrence of agitation and command-following (days)**	***n*** **= 119**	
≤3	*n* = 54 (44.6%)	
≤7	*n* = 81 (67.8%)	
≤14	*n* = 96 (80.7%)	
**Correlation**	**Coefficient**	***P*** **value**
Agitation and command-following	0.315	**<0.001**
Time to agitation and time to command-following	0.485	**<0.001**

*Bold texts highlight the statistical significance*.

### Antipsychotic Use in Agitated Patients Does Not Affect Clinical Outcomes

Seventy-five (47.2%) patients with agitation received antipsychotic medication as a treatment for agitated symptoms, and 32 of them received more than one type of antipsychotics. Haloperidol was the most commonly administered antipsychotic medication (*n* = 43, 57.3%), followed by quetiapine (*n* = 39, 52.0%). Other antipsychotics included olanzapine (*n* = 26, 34.7%), risperidone (*n* = 6, 8.0%), ziprasidone (*n* = 1, 1.3%) and aripiprazole (*n* = 1, 1.3%). Baseline patient characteristics, such as age, sex, admission GCS, ISS, or Rotterdam score, were not different between patients who received antipsychotics and those who did not (Table 4). There were no significant differences in LOS (34.5 vs. 28.7 days, *p* = 0.16), TFC (9.6 vs. 10.7 days, *p* = 0.55), discharge GCS (*p* = 0.13), or discharge location (*p* = 0.19) between patients who received antipsychotics vs. those who did not (Table 4). Administration of multiple types of antipsychotics compared to a single type or the use of haloperidol did not correlate with a change in LOS, TFC, discharge GCS, or discharge location.

**Table 4:** Comparing patient characteristics and clinical outcomes between patients who received antipsychotics for agitation vs. those who did not receive antipsychotics, *n* = 159.

**Patient characteristics**	**Received antipsychotics (*n* = 75, 47.2%)**	**Did not receive antipsychotics (*n* = 84, 52.8%)**	***P*-value**
Mean Age (Years)	39.3 (SD 17.3)	41.9 (SD 17.5)	0.35
Sex			
Female	16 (50.0%)	16 (50.0%)	0.72
Male	59 (46.5%)	68 (53.5%)	
Mean GCS on admission	4.7 (SD 1.9)	4.8 (SD 1.9)	0.61
Mean ISS	29.5 (SD 13.9)	26.3 (SD 12.6)	0.12
Mean Rotterdam	2.9 (SD 0.9)	2.8 (SD 0.9)	0.81
**Patient outcomes**	**Received antipsychotics**	**Did not receive antipsychotics**	
Mean LOS (Days)	34.5 (SD 28.3)	28.7 (SD 22.3)	0.16
95% confidence interval for mean	27.8–41.1	23.8–33.5	
Median	25.5	21.5	
Mean TFC (days)	9.6 (SD 7.7)	10.7 (SD 13.0)	0.55
95% confidence interval for mean	7.8–11.4	7.8–13.5	
Median	8.0	5.5	
Discharge GCS	No. (%)		
13–15	72 (96.0%)	75 (86.2%)	0.13
9–12	1 (1.3%)	7 (9.2%)	
3–8	2 (2.7%)	2 (4.6%)	
Discharge location	No. (%)	No. (%)	
Home	19 (25.3%)	30 (35.7%)	0.19
Rehabilitation	51 (68.0%)	50 (59.5%)	
Nursing home or hospice	1 (1.3%)	4 (4.8%)	
Other hospital	4 (5.3%)	0 (0.0%)	

*GCS, Glasgow Coma Scale; ISS, Injury severity score; LOS, Length of hospital stay; TFC, Time to follow commands*.

## Discussion

The present study is by far the largest series reporting the association between posttraumatic agitation and recovery of consciousness during early stage of sTBI in adult patients. Our data shows that agitation often accompanies the ability to respond to verbal commands and can be viewed an early sign of neurological recovery. Among all 169 patients who developed agitation, 162 (95.5%) survived, and 159 (94.1%) followed commands. This result suggests that posttraumatic agitation strongly correlates with command-following. Thus, in minimally conscious patients, agitation can be viewed as a positive sign that these patients are more likely to “wake up” and exhibit more meaningful signs of neurological recovery. Following severe trauma, the brain undergoes rapid biochemical, hormonal, and structural network remodeling (9), and these processes may manifest as agitated behaviors. Our results show that agitated patients often have better clinical outcomes at hospital discharge compared to non-agitated individuals. A higher percentage of agitated patients were discharged with GCS 13–15; agitated patients were more frequently discharged home or to rehabilitation facilities instead of nursing home or hospice. This difference may be partly explained by the younger age of the agitated group seen in our analysis. The length of hospital stay (LOS) and time to follow commands (TFC) did not differ between the agitated and the non-agitated groups. Although agitated behaviors pose challenges to medical care, their presence appears to reassure favorable prognoses.

The etiology of agitated behaviors in critically ill patients following sTBI is multifactorial (26), Pain and infection, for example, cause systemic catecholamine dysregulation and psychomotor disturbance. In comatose patients emerging from disorders of consciousness, being able to regain awareness to immediate surrounding is a sign of arousal (27). As patients undergo further neurological recovery, they develop the ability to interfere with medical treatment to minimize discomfort. The reemergence of these goal-directed behaviors, clinically present as severe agitation, strongly suggests the recovery of consciousness (27). In the sTBI population, continuous sedation is administered due to the need for mechanical ventilation, and clinical improvement prompts discontinuation of these interventions. Interestingly, agitation is often observed during these periods, as patients demonstrate non-purposeful movements and self-harming behaviors when recovering from coma. Attempts to disrupt patient-ventilator synchrony to minimize discomfort is an example of early goal-directed behavior that indicates one's awareness of immediate surroundings. These behaviors are highly suggestive of the potential for more complex and meaningful recovery of higher-level neurological functions.

Clinical management of agitation in sTBI patients consists of environmental changes and adequate sedation to minimize patient discomfort (14). When these conservative measures fail, antipsychotics are the mainstay of treatment for agitation. In our study, nearly half of agitated patients received antipsychotics. A previous study suggests that antipsychotics have neuroprotective effects in critically-ill TBI patients, and thus improve in-hospital outcomes (28). In the present study, the use of antipsychotics did not correlates with LOS, TFC, discharge GCS, or discharge location. These outcomes were also not different between groups that received multiple types vs. single type, or those who received typical vs. atypical antipsychotics. Despite that antipsychotics may reduce symptoms of agitation in acute settings (29), its long-term effects on overall cognitive functions have not been thoroughly investigated (30, 31). Moreover, suppression of dopaminergic neurotransmission can be unfavorable for posttraumatic arousal as clinical trials in amantadine have shown efficacy (12). The ongoing randomized controlled clinical trials using antipsychotics to treat posttraumatic agitation in subacute rehabilitation settings may shed light on the management of emotional dysregulation as a result of sTBI (23, 24). The treatment of posttraumatic agitation in acute hospital setting requires further large-scale prospective randomized studies with long-term follow up. When managing agitated symptoms, clinicians should bear in mind that they are often a positive clinical sign that indicates a vibrant state of recovering consciousness.

Finally, posttraumatic agitation was found in 31.9% of our total study population, and 95.9% of these patients survived. The agitated cohort was on average 8.6 years younger than the non-agitated cohort. This result is consistent with an observational study conducted in a trauma intensive care unit that investigated all types of traumatic injuries (32). Additionally, we found that a larger proportion of male patients developed posttraumatic agitation compared to females. Sex hormones are recognized as key modulators of the pathogenic process after TBI by affecting brain metabolism and neural repair (33). During the early phase after TBI, neuroendocrine dysregulation alters the ratios of estrogen, progesterone, and testosterone. This process plays an important role in TBI recovery and rehabilitation (34). Further research is required to understand these molecular and biochemical mechanisms in order to develop effective treatments that facilitate both short-term recovery of consciousness and long-term cognitive functioning.

Future studies using advanced neuroimaging have the potential to reveal patient-specific factors and aberrant neural activity that predispose sTBI patients to posttraumatic agitation. In our study, 41.5% of patients recovered consciousness but did not develop agitation. This result may be explained by patient-specific characteristics and/or lesions to specific locations or neural network. Previous studies conducted in patients with penetrating TBI suggest that lesions in the prefrontal cortex are highly predictive of long-term agitation and aggression (35). Identification of neural correlates of agitation in sTBI patients can facilitate the development of therapeutic strategies for treating these disruptive behaviors.

## Limitations

There are several limitations to our study. First, this study is based on a retrospective chart review of adults (≥18 years) with a diagnosis of sTBI (GCS ≤ 8). The results only infer associations but not causal relationships within this population. Second, in our study, the Richmond Agitation Sedation Scale (RASS) and daily clinical assessment were used to diagnose agitation. Other measures, such as the Agitated Behavior Scale, use different criteria to characterize agitation and delirium when compared with the RASS. This makes comparisons with other studies difficult. Third, injury site was not included in the analysis. Many patients suffered injuries at various locations and subcategorizing patients to isolate the effects of injury site was difficult. Finally, the return of consciousness was defined as the ability to follow motor commands. Some patients may recover from comatose state, yet, be unable to consistently follow commands due to neuromuscular insufficiency (36). Outcome data were limited to the hospital stay only, and the authors cannot comment on the impact of the above factors on any longer-term outcome measures. Future prospective studies with long-term follow up are needed to reveal the underlying neurological mechanisms of in-hospital agitation in sTBI patients to better understand its clinical indication and to provide appropriate management.

## Conclusion

Severe traumatic brain injury (sTBI) commonly results in disorders of consciousness. This study suggests that the presence of agitation during the early stage after sTBI is a clinical sign associated with command-following and is associated with recovery of consciousness. Additionally, patients with agitation often demonstrate more favorable clinical outcomes. Management of agitation should be based on the consideration of patient characteristics, risk and benefit of pharmacological intervention, and effects on long-term cognitive functioning. Using antipsychotics to reduce agitated symptoms does not affect in-hospital outcomes, but its effects on neurocognitive recovery remain inconclusive. Clinicians should bear in mind that agitation is a sign of recovery of consciousness when treating this condition and counseling families.

## Data Availability Statement

The data supporting the conclusions of this article will be made available by the authors at reasonable request.

## Ethics Statement

The studies involving human participants were reviewed and approved by Stony Brook University Committee on Research in Human Subjects. Written informed consent for participation was not required for this study in accordance with the national legislation and the institutional requirements.

## Author Contributions

ZW, NW, ZZ, and MC collected data. ZW completed statistical calculations and wrote the manuscript. NW, CM and SM assisted in study design and critically revised the manuscript. TG, JS, and RM revised the manuscript. SF managed the database. CM and SM provided overall supervision. All authors contributed to the article and approved the submitted version.

## Conflict of Interest

The authors declare that the research was conducted in the absence of any commercial or financial relationships that could be construed as a potential conflict of interest.
